# Does hyperbaric oxygen therapy truly help in the treatment of chronic recurrent multifocal osteomyelitis?

**DOI:** 10.1186/1546-0096-12-S1-P263

**Published:** 2014-09-17

**Authors:** Barbara Kvenić, Vojko Rožmanić, Aleksandar Ovuka

**Affiliations:** 1 Clinical Hospital Center Rijeka, Rijeka, Croatia

## Introduction

Chronic recurrent multifocal osteomyelitis (CRMO) is a non – infectious inflammatory bone disorder of yet to be determined etiopatology. However, it is considered that the imbalance between pro- and anti- inflammatory cytokines plays the main role in the disease expression. CRMO represents the most severe form of chronic nonbacterial osteomyelitis and predominantly affects children and adolescents, primary girls. The clinical presentation is variable onset of bone pain with or without fever, local soft tissue swelling or warmth with an unpredictable clinical course and spontaneous remissions. Methaphyses of long bones are the most specific affected sites. CRMO is a diagnosis of exclusion because of its clinical presentation and laboratory findings that are not disease-specific and vary between patients.

## Objectives

Here we present two cases of CRMO, first in 9-year-old girl localized in left clavicle and the second one in a 5-year-old girl with oligo-focal form. In both cases onset of the disease was characterized with bone pain and local soft tissue swelling without fever. Laboratory findings included elevated inflammatory markers (C-reactive protein (CRP), erythrocyte sedimentation rate (ESR), leucocytes count). Radiographs, bone scan and MRI in both girls showed specific signs indicating osteomyelitis (osteolytic lesions located in the metaphyses, nonagressive periostal reaction, thickening of surrounding soft tissue). A needle biopsy was performed and the pathohistological findings indicated osteomyelitis. Microbiological findings were negative. Both patients were initially treated by antibiotic. During the first week of antibiotic therapy older girl was developed DRESS – drug rash with eosinophilia and systemic symptoms. The recurrence of the symptoms, multifocality, lack of evidence of causative factor and imaging characteristics of the bone lesions led us to CRMO diagnosis. In both girls we applied treatment with NSAID and corticosteroids (prednisone 1 mg/kg for two weeks with subsequent reducing of dosage for four weeks). In the same time both patients were subjected to hyperbaric oxygen therapy.

## Methods

In the same time both patients were subjected to hyperbaric oxygen therapy. After six months clinical remission was achieved in the both children but with residual bone impairments on MRI scans.

## Results

After six months clinical remission was achieved in the both children but with residual bone impairments on MRI scans.

## Conclusion

Since CRMO is self limited and benign disease the question is whether oxygen barotherapy truly helps or is it natural course of the disease. According to the literature data residual bone impairments are expected, which suggest an up to 50% rate of residual impairments.Thus, the development of effective treatment algorithm and enlightened of pathofisiology of CRMO will be essential in the future.

## Disclosure of interest

None declared.

